# Supplementary material for: Exploring Novel 2D Analogues of Goldene: Electronic, Mechanical, and Optical Properties of Silverene and Copperene
Source: ACS Omega. 2025 Jun 17;10(25):26892–900. doi: 10.1021/acsomega.5c01823 (PMC12223819; doi:10.1021/acsomega.5c01823)
Supplement: Supplementary file 1 [file ao5c01823_si_001.pdf]

Supplementary Information: Exploring Novel 2D Analogues of Goldene: Electronic, Mechanical, and Optical Properties of Silverene and Copperene

Emanuel J. A. dos Santos,[1,2] Rodrigo A. F. Alves,[1,2] Alexandre C. Dias,[3]
Marcelo L. Pereira, Jr,[4] Douglas S. Galvão,[5] and Luiz A. Ribeiro, Jr[1,2]

[1] *University of Brasília, Institute of Physics, 70910-900, Brasília, Federal District, Brazil.*

[2] *Computational Materials Laboratory, LCCMat, Institute of Physics, University of Brasília, 70910-900, Brasília, Federal District, Brazil.*

[3] *Institute of Physics and International Center of Physics, University of Brasília, 70919-970, Brasília, Federal District, Brazil.*

[4] *University of Brasília, College of Technology, Department of Electrical Engineering, 70910-900, Brasília, Federal District, Brazil.*

[5] *Department of Applied Physics and Center for Computational Engineering and Sciences, State University of Campinas, 13083-859, Campinas, São Paulo, Brazil.*

*Corresponding author: `ribeirojr@unb.br`

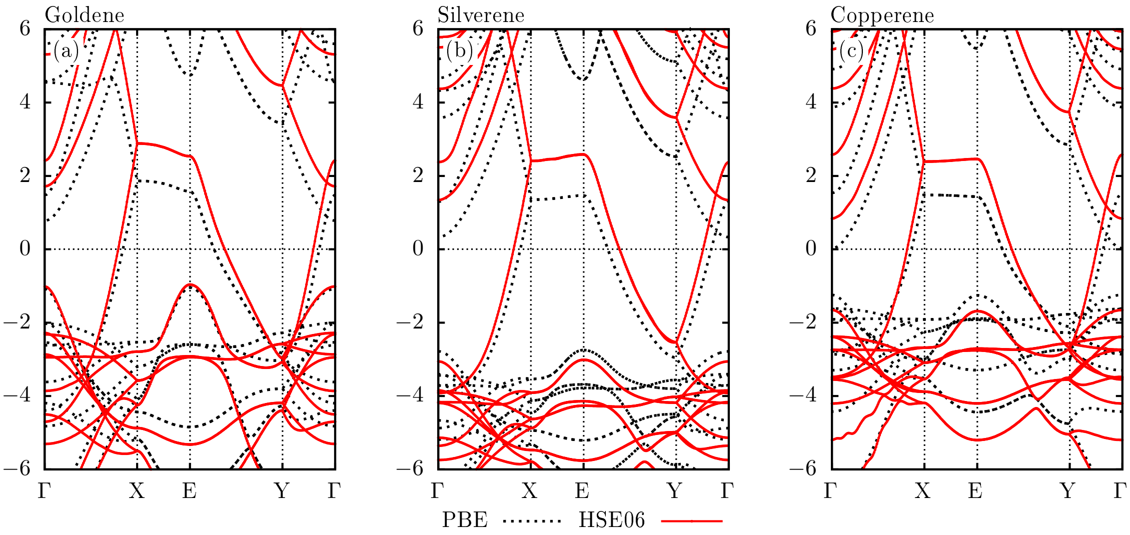

FIG. S1. Electronic band structures of (a) Goldene, (b) Silverene, and (c) Copperene calculated using PBE (black dotted lines) and HSE06 (red solid lines). The high-symmetry path is given by Γ–X–E–Y–Γ, and the zero of energy is set at the Fermi level.
